# ‘Syntactic Perturbation’ During Production Activates the Right IFG, but not Broca’s Area or the ATL

**DOI:** 10.3389/fpsyg.2016.00241

**Published:** 2016-02-23

**Authors:** William Matchin, Gregory Hickok

**Affiliations:** ^1^Cognitive Neuroscience of Language Laboratory, Department of Linguistics, University of Maryland, College ParkMD, USA; ^2^Auditory and Language Neuroscience Laboratory, Department of Cognitive Sciences, University of California, Irvine, IrvineCA, USA

**Keywords:** syntax, sentence processing, language, fMRI, inferior frontal gyrus, Broca’s area, ATL, production

## Abstract

Research on the neural organization of syntax – the core structure-building component of language – has focused on Broca’s area and the anterior temporal lobe (ATL) as the chief candidates for syntactic processing. However, these proposals have received considerable challenges. In order to better understand the neural basis of syntactic processing, we performed a functional magnetic resonance imaging experiment using a constrained sentence production task. We examined the BOLD response to sentence production for active and passive sentences, unstructured word lists, and *syntactic perturbation*. Perturbation involved cued restructuring of the planned syntax of a sentence mid utterance. Perturbation was designed to capture the effects of syntactic violations previously studied in sentence comprehension. Our experiment showed that Broca’s area and the ATL did not exhibit response profiles consistent with syntactic operations – we found no increase of activation in these areas for sentences > lists or for perturbation. Syntactic perturbation activated a cortical-subcortical network including robust activation of the right inferior frontal gyrus (RIFG). This network is similar to one previously shown to be involved in motor response inhibition. We hypothesize that RIFG activation in our study and in previous studies of sentence comprehension is due to an inhibition mechanism that may facilitate efficient syntactic restructuring.

## Introduction

Language can be analyzed as a cognitive faculty consisting of several components, including a core structure-building system – *syntax* – that operates over stored lexical atoms (Chomsky, 1982, 1995; Hauser et al., 2002). Much work attempting to localize syntactic operations has focused on Broca’s area (Stromswold et al., 1996; Hagoort, 2005; Grodzinsky and Santi, 2008; Friederici, 2011) in the left inferior frontal gyrus (LIFG; Brodmann areas 44 and 45) and the anterior temporal lobe (ATL; Rogalsky and Hickok, 2009; Bemis and Pylkkänen, 2011; Brennan et al., 2012). However, the response profile of Broca’s area during sentence comprehension appears to be more compatible with a domain-general function such as working memory or cognitive control than with syntax (Kaan and Swaab, 2002; Novick et al., 2005; Rogalsky and Hickok, 2011; Bornkessel-Schlesewsky and Schlesewsky, 2013), although this is still a hotly debated issue (Hickok and Rogalsky, 2011; Fedorenko et al., 2012b). Similarly, recent neuroimaging and neuropsychological studies have implicated the ATL in semantic rather than syntactic processes (Rogalsky and Hickok, 2009; Pallier et al., 2011; Del Prato and Pylkkanen, 2014; Wilson et al., 2014).

We chose to perform a functional magnetic resonance imaging (fMRI) experiment during sentence production to contribute to this debate. Sentence production studies in fMRI and magnetoencephalography (MEG) have revealed large overlap with the activation patterns found in comprehension, suggesting that similar neural networks underlie sentence processing in both modalities (Braun et al., 2001; Blank et al., 2002; Haller et al., 2005; Golestani et al., 2006; Menenti et al., 2011; Segaert et al., 2012; Del Prato and Pylkkanen, 2014; Pylkkänen et al., 2014). These studies are informative with respect to the neurobiology of sentence production; however, few production studies have manipulated syntactic variables compared to the vast literature on syntactic processing in comprehension. Syntax production studies will be important to provide complementary evidence to the comprehension literature to better understand syntactic processing in the brain.

We attempted to parallel the effects of syntactic violations that several researchers have used to study syntax in comprehension (e.g., Embick et al., 2000; Moro et al., 2001; Friederici et al., 2003). However, there are significant obstacles in extending the violation approach to production. Instructing subjects to produce artificial syntactic errors means that subjects will expect the upcoming violation. This may eliminate the effect of interest because expectation lessens the strength of the neural response to syntactic violations (Lau et al., 2006). The short time constraints of fMRI make difficult capturing infrequent natural errors or using a paradigm to induce subjects to produce them (Ferreira and Swets, 2005). Because of these reasons, we forced subjects to intermittently and unexpectedly switch their planned syntactic structure mid-utterance. The logic is that switching structures increases demands on the neural resources involved in syntactic processing (as well for other mechanisms). We expected to capture this effect in the blood-oxygen level-dependent (BOLD) response during scanning.

In the present study, we controlled the syntactic structure of the subjects’ utterances with a constrained sentence elicitation task, similar to Caplan and Hanna (1998). To induce syntactic restructuring, we borrowed the target perturbation paradigm from motor control research (Paulignan et al., 1991; Elliott et al., 1995; Izawa et al., 2008). In this paradigm subjects attempt to hit a target, e.g., reaching from one point to another on a screen. On most trials the subject’s target and/or sensory feedback remain constant throughout the trial. On a smaller proportion of random trials, the subject’s target or sensory feedback is altered mid-movement. For example, the target location changes, or a force is applied to the subject’s arm. On such trials, the subject must adapt and correct the movement trajectory online to reach the goal. We adapted this approach to syntax, dubbing our paradigm “syntactic perturbation.”

We trained subjects to produce either active sentences (e.g., *Susan is following Charlie*) or passive sentences (e.g., *Charlie is being followed by Susan*). On most trials (standard trials, 80%) subjects did not switch their planned structure. On a smaller proportion of random trials (switch trials, 20%) a cue prompted subjects to switch structures mid-utterance. In other words, on switch trials the cue prompted the subject to switch from active to passive or from passive to active. This task was artificial, raising questions about the ecological validity of our experiment – such considerations should be kept in mind when evaluating the results. However, we assumed subjects would update their syntactic structure regardless of this artificial nature.

The key assumptions of our experiment are the following: (1) the planned syntactic structure of an utterance is built in advance of speech production (at least for mono-clausal active and passive sentences), and (2) this plan can be dynamically updated during speech production. The first assumption is supported by the fact that juxtaposition errors often occur for words or phrases of the same syntactic category and from the same syntactic position (Fromkin, 1971) – to account for this regularity, speakers must have built the syntactic structure in advance of articulation. The second assumption is supported by an experiment showing that speakers decrease their rate of speech predictively if the structure they ultimately utter contains a syntactic violation (Ferreira and Swets, 2005).

Our design consisted of two main contrasts: STRUCTURE (sentences > word lists) and PERTURBATION (switch trials > standard trials), and one secondary contrast: COMPLEXITY (passive sentences > active sentences). Using our novel paradigm in production rather than comprehension, we examined the response profile of these contrasts in areas traditionally associated with syntax. Our main goal was to further inform the debate on the role of these regions in syntactic processing. Also, we believe that the discussion of the neurobiology of syntax has focused overwhelmingly on the ATL and Broca’s area because of the repeated use of similar experimental manipulations in comprehension. We sought to determine whether our experiment, differing in modality and task, found activation in areas outside of these regions, potentially indicating a role for them in syntactic processing. We discuss our predictions for each of these contrasts in turn.

The contrast of sentence > word lists in comprehension has frequently revealed activation in the ATL, often bilaterally but also left lateralized (e.g., Mazoyer et al., 1993; Humphries et al., 2005; Rogalsky and Hickok, 2009). Two recent MEG studies found increased activation for preparation to produce two-word phrases compared to production of single words (Pylkkänen et al., 2014) and two-word lists (Del Prato and Pylkkanen, 2014). Current research supports a semantic interpretation of ATL function that drives these effects. We expected that the contrast of STRUCTURE in our study would also activate the ATL because the production of sentences presumably requires semantic processing that the production of lists does not.

The sentence > list contrast in comprehension occasionally activates Broca’s area (Bedny et al., 2011; Fedorenko et al., 2011; Pallier et al., 2011), but these effects are much less consistent than for the ATL (Rogalsky and Hickok, 2011). This suggests that this activation reflects the contribution of working memory resources or cognitive control mechanisms needed to parse difficult input rather than fundamental syntactic operations (Novick et al., 2005; Rogalsky et al., 2008). Our sentences are short, simple in structure, and guided by a strict template; we believe this minimizes demands on these mechanisms. Therefore we did not expect STRUCTURE to activate Broca’s area.

For similar reasons, we also expected that Broca’s area would not show a significant effect of PERTURBATION. During switch trials, subjects had an unambiguous selection of the alternative sentence construction, which should minimize selection demands (Miller and Cohen, 2001). With respect to working memory, these resources are taxed in conditions that place heavy demands on maintenance of information or retrieval across intervening material (Baddeley, 1992; Gibson, 2000; Lewis et al., 2006). During switch trials, subjects had to quickly restructure their utterance but did not have to maintain additional material or retrieve information across long distance.

We did not have equally strong predictions for the PERTURBATION contrast in the ATL. Although the existing evidence does not support a role for the ATL in syntax, changes in syntactic structure lead to changes in semantic interpretation (Chomsky, 2014). If the ATL plays a role in combinatory semantics, syntactic restructuring might induce activation in this region for semantic processes. Therefore we expected to potentially see increased activation for PERTURBATION in the ATL.

Significant effects of PERTURBATION outside these areas could reflect syntactic operations in areas not traditionally associated with syntax, such as subcortical areas (see Lieberman, 2001; Ullman, 2004; Boeckx and Benítez-Burraco, 2014 for these non-standard proposals). Activation for this contrast could also reflect non-syntactic mechanisms. These could be linguistic (e.g., reanalysis of thematic role assignment), or non-linguistic (e.g., error detection, attention). In the discussion section we discuss robust activation of the right IFG for PERTURBATION in the context of the literature on action inhibition and the role it may play in syntactic restructuring.

The secondary COMPLEXITY contrast (passive/complex > active/simple) is an extension of previous work that has shown increased activation in Broca’s area for passive sentences (e.g., Ye and Zhou, 2009; Mack et al., 2013). The standard interpretation of this finding is that increased syntactic processing resources are used to process passive sentences. However, while historical approaches of generative grammar (Chomsky, 1982, 2002, 2014) posited a syntactic complexity difference between passives and actives (application of a movement operation in passives), modern syntactic theory does not (largely due to the VP-internal subject hypothesis – active sentences also involve movement of the subject, Kitagawa, 1994). Any complexity difference between passives and actives therefore likely lies in non-syntactic factors, such as the mapping of arguments to thematic roles (we thank an anonymous reviewer for pointing this out). The fact that Broca’s area *does* show increased activation for passives during comprehension supports a non-syntactic interpretation of the function of this region. Whether this region shows increased activation for passive compared to active sentences during production is an open question and should inform hypotheses of this region’s function. We did not have strong predictions for this contrast, but included it because it allowed comparison with previous research in comprehension.

## Materials and Methods

### Subjects

Twenty-one right-handed, native speakers of English (age 19–33, 10 female) volunteered for participation. Subjects had normal or corrected-to-normal vision, no hearing impairment, and reported no history of neurological disorder. Subjects were paid $10 for participation in a 1-hour behavioral training session. One subject was excluded from the fMRI portion of the experiment due to difficulty with the task during the behavioral session resulting in 20 remaining subjects in the fMRI experiment. Subjects were paid $30 an hour for participation in the fMRI session. Consent was acquired from each subject before participation and all procedures were approved by the Institutional Review Board of UC Irvine.

### Stimuli

**FIGURE 1:**
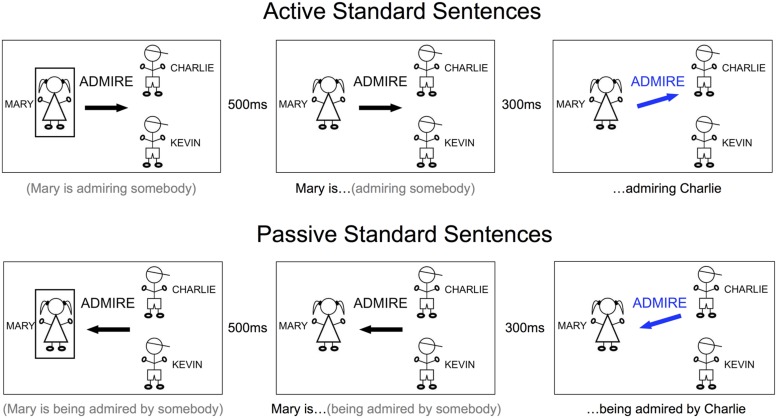
**Sample trials for standard sentences, active and passive.** The words under each picture indicate what the subject was able to plan (in gray) and what the subject produced or began to produce (in black) during that stage of the cue. Only the image within the large black rectangles was part of the stimulus. LEFT: PREP stage, in which the subject prepared to begin producing the sentence with either active or passive construction. MIDDLE: GO stage, in which subject was cued to begin producing the incomplete sentence. RIGHT: FINISH stage, in which the completing information was presented. On standard trials, the subject’s final structure was consistent with planning.

The stimulus for each trial consisted of a cue that progressed through three stages: PREP, GO, and FINISH. Every stimulus had the same basic appearance: simple line drawings of the people engaged in the target sentence, the names of the people in large font next to the drawings, the verb to be used in the sentence in the middle of the screen, and an arrow underneath the verb pointing to the right or the left (**Figure 1**). Identical stimulus presentation was used for both sentences and lists – only the subject’s task changed. Twenty different transitive verbs were used. Verb length varied from one to three syllables. Verbs were selected for a mix of articulatory complexity. Here is the complete list of verbs: admire, deceive, examine, follow, frighten, greet, harass, help, hug, kick, kiss, pinch, poke, protect, punch, push, rob, scare, tease, tickle.

Verbs were randomly distributed throughout the experimental runs. Four people were used with these names: Mary, Susan, Charlie, and Kevin. The first person was always a different gender than the final person, and people were randomly distributed in different positions throughout the experimental runs. Three people appeared on each cue: one person on the left (START) and two on the right (END). The END people were displayed vertically, one above the other. During the first stage, the PREP stage, a rectangular box surrounded the START person. The arrow, in black color, pointed from the START person horizontally toward the middle of the END people, and not directly toward either of them. During the second stage, the GO stage, the rectangular box disappeared, serving as a “go” signal for the subject to begin articulating. During the third stage, the FINISH stage, the arrow turned blue and tilted up or down to point to the target END individual for that trial. This design forced the subject to begin articulating without knowing which person to end the sentence with and to use the information provided at the FINISH stage to complete the sentence with the correct person. The PREP stage lasted for 500 ms. We chose this time to give subjects enough time to process the information and plan their utterances. The GO stage began immediately after the 500 ms, and subjects began articulating in synchrony with the disappearance of the box. The interval between the GO stage and the FINISH stage was 300 ms, and the FINISH stage remained on the screen for 1000 ms, followed by fixation until the next trial. During the behavioral training session, the subject would initiate the next trial whenever ready. During the fMRI session, the inter-trial-interval was fixed at 4200 ms, for a total trial duration/inter-trial interval of 6 s.

### Task

The task was production of either sentences or lists and to restructure appropriately to the switch cue. This resulted in a 2 × 2 design: **STRUCTURE** (sentence, list) and **PERTURBATION** (standard, switch). In the sentence condition, subjects produced sentences with either active or passive construction using the template detailed below. These two constructions comprised an additional sub-factor within the sentence condition, **COMPLEXITY** (active/simple, passive/complex). Active sentences were cued with an arrow pointing away from the first person, and passive sentences were cued with an arrow pointing toward the first person (**Figure 1**). Active sentences were produced with this template: *(person 1)* ***is*** *(verb)****ing*** *(person 2).* e.g., *Mary is following Charlie*. Passive sentences were produced with this template: *(person 1)* ***is being*** *(verb)****ed by*** *(person 2)*. e.g., *Mary is being followed by Charlie*. We instructed subjects to use the progressive aspect on every trial and not to deviate from the template. In the list condition, subjects produced a list of words based on the information from the cue. Subjects ignored the identity of the particular verb on the cue and did not use it in their lists. When the arrow pointed to the right (as in active sentences), subjects produced a list with this template: *(person 1) “word* ***right*** *arrow” (person 2)*, .e.g., *“Mary word right arrow Charlie.”* When the arrow pointed to the left (as in passive sentences), subjects produced a list with this template: *(person 1) “word* ***left*** *arrow” (person 2)*. e.g., *“Mary word left arrow Charlie.”* We chose the word *word* to approximately control for the duration of planning and articulation that would take place for the word *is* in the sentence condition. This timing was relevant to when the subjects were cued to restructure during their utterance (we discuss this in more detail below). Subjects made their utterances at a natural speaking rate.

Subjects did not know how to complete the sentence/list at the beginning of each trial. The FINISH stage indicated which person (top or bottom) would be the second person in the sentence. Subjects were instructed to begin their utterances at the GO stage and use the information on the FINISH stage to determine which name to produce. We set the ISI between the GO stage and the FINISH stage to be 300 ms to allow subjects enough time while speaking naturally to update their utterance without making mistakes on the switch trials. As an example, if the target sentence were “Mary is following Charlie,” at the GO stage subjects started speaking “Mary is following …”, then 300 ms later at the FINISH stage they updated their plan to include “Charlie” and finished. Similarly for the list condition, if the target list were “Mary word left arrow Charlie,” they would start speaking “Mary word left arrow…” at the GO stage, and update at the FINISH stage to include “Charlie.”

**FIGURE 2:**
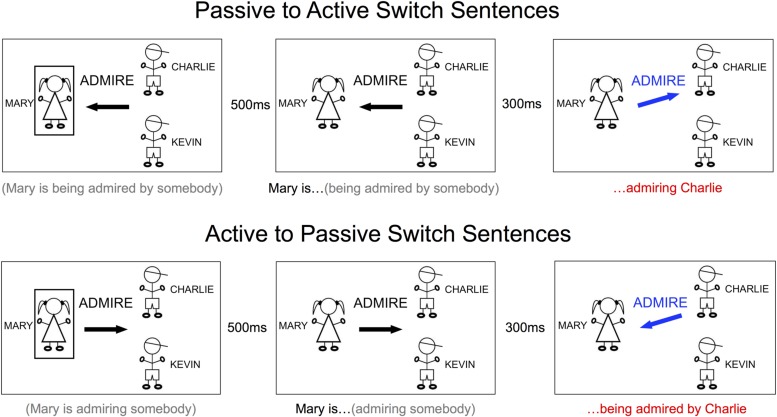
**Sample trials for switch (perturbation) sentences, passive to active and active to passive.** The words under each picture indicate what the subject was able to plan (in gray) and what the subject produced, or began to produce (in black), and what the subject produced after updating the sentence construction (in red) when that cue was presented. Only the image within the large black rectangles was part of the stimulus. LEFT: PREP stage, during which the subject prepared to begin producing the sentence with either active or passive construction. MIDDLE: GO stage, during which subject was cued to begin producing the incomplete sentence. RIGHT: FINISH stage, during which the completing information was presented. On switch trials, the subject would have to change from one structure to another.

Standard trials occurred as described above; switch trials involved not only updating person 2, but also switching the orientation of the arrow mid-production (**Figure 2**). On sentence switch trials, subjects switched their target sentence from active to passive or vice versa, e.g., *Mary is following (person 2)* →*Mary is* ***being followed by Charlie.*** During list switch trials, subjects needed to switch whether they said *right arrow* or *left arrow*, e.g., *Mary word left arrow (person 2)* → *Mary word* ***right*** ***arrow Charlie.*** Standard and switch trials were presented at a 4/1 ratio and in random order within each run, such that subjects could not predict what the next trial would be. We used this ratio because this approximate ratio was used in previous studies of target perturbation and fMRI (3/1 ratio used by Tourville et al., 2008), and a smaller ratio of standard to switch trials might have resulted in anticipation of switch trials. We did not want subjects to use a strategy of not committing to a syntactic plan on every trial in order to avoid errors.

The sentence and list conditions were presented in separate runs to avoid confusion and task-switching effects. To balance the spatial orientation of the cues, we counterbalanced across sides by presenting subjects with cues that flowed from left to right (depicted in **Figures 1** and **2**) and cues that flowed from right to left (active sentences correspondingly began with a left arrow instead of a right arrow). Subjects always received two runs from either the sentence or list condition in a row, one each of left and right cue orientation (order counterbalanced across subjects), and we collapsed all analyses across the two orientations.

### Behavioral Training Session

Before running the experiment in the fMRI scanner, we familiarized subjects on the task in a behavioral training session. We wanted subjects to be well prepared for the task in the fMRI scanner to limit variance in performance as well as minimize effects of exposure. In the training session we explained the task to the subjects, including a demonstration by the experimenter on several trials. Then, subjects were asked to perform the task themselves. In the first several trials, the experimenter remained in the testing room to give feedback and instruction. When the subject grasped the task, the experimenter left the room and the subject proceeded self-paced. Subjects performed both tasks with both orientations for a total of four experimental runs, consisting of 50 trials apiece, for a total of 100 trials in the sentence condition and 100 trials in the list condition. The subjects’ utterances were recorded and their performance was analyzed. A subject’s response was considered an error if they produced the incorrect sentence construction (e.g., active instead of passive), produced the word *right* instead of the word *left* (or vice versa), or if they made a speech error during the trial (e.g., produced the wrong speech sound, extensive delays, etc.). Substituting the names of people (e.g., *Mary* instead of *Susan*) or substituting one verb for another (e.g., *push* instead of *punch*) were not counted as errors, unless the subject also made an additional error as described above. We were only able to collect and analyze behavioral data from 14 out of 20 subjects due to equipment issues. To assess the effect of perturbation on behavior, we averaged across constructions in the sentence conditions and direction in the list conditions. We then performed a 2 × 2 ANOVA (STRUCTURE × PERTURBATION). Subjects underwent the fMRI portion of the experiment after completing the behavioral session, either the same day or on a subsequent day, within a week after the behavioral session.

### fMRI Experiment

Before scanning, subjects were briefly re-familiarized with the task by performing a few trials in each condition outside the scanner. Subjects were instructed to produce their utterances out loud in the scanner, but quietly and with minimal articulation. Subjects received 12 total experimental runs during the experiment (six sentence, six list, counterbalanced by orientation). During the experiment, a fixation cross was displayed on a screen in-between trials. Stimuli were delivered with Matlab software (Mathworks, Inc, USA) utilizing Psychtoolbox (Brainard, 1997; Kleiner et al., 2007). Subjects were given ear covers and foam earplugs to attenuate scanner noise. Each run contained 40 standard trials and 10 switch trials in random order with no explicit rest trials. Presentation order of sentence and list runs was counterbalanced along with cue orientation across subjects. Active/passive constructions and left/right arrow lists were presented at equal frequency. The high-resolution anatomical image was collected following the experimental runs. The scanning session lasted about 1 h and 15 min in total.

### fMRI Data Collection and Analysis

MR images were obtained in a Philips Achieva 3T (Philips Medical Systems, Andover, MA, USA) fitted with an eight-channel RF receiver head coil at the high field scanning facility at UC Irvine. We first collected a total of 1896 T2*-weighted EPI volumes over 12 runs using Fast Echo EPI in ascending order (*TR* = 2 s, *TE* = 25 ms, flip angle = 90°, in-plane resolution = 1.95 mm × 1.95 mm, slice thickness = 3 mm with 0.5 mm gap). The first four volumes of each run were collected before stimulus presentation and discarded to control for T1 saturation effects. The high-resolution T1-weighted anatomical image was acquired in the axial plane (*TR* = 8 ms, *TE* = 3.7 ms, flip angle = 8°, size = 1 mm isotropic).

Slice-timing correction, motion correction, and spatial smoothing were performed using AFNI software (http://afni.nimh.nih.gov/afni). Motion correction was achieved by using a 6-parameter rigid-body transformation, with each functional volume in a run first aligned to a single volume in that run. Functional volumes were aligned to the anatomical image, and subsequently aligned to Talairach space (Talairach and Tournoux, 1988). Functional images were resampled to 2.5 mm isotropic voxels and spatially smoothed using a Gaussian kernel of 6 mm FWHM. Finally, functional images were rescaled to reflect percent signal change from the mean signal during each run.

First-level analyses were performed on each individual subject’s data using AFNI’s 3dDeconvolve function. The regression analysis was performed to find parameter estimates that best explained variability in the data. Each predictor variable representing the time course of activity associated with the task was entered into a deconvolution analysis that estimated parameters best representing the timecourse of the hemodynamic response function in percent signal change values. Timecourse estimates were modeled beginning with the onset of the PREP stage, i.e., when the subject began planning the sentence. The following eight regressors of interest were used in the experimental analysis: sentence active, sentence passive, list left, list RIGHT, SENTENCE SWITCH: ACTIVE TO PASSIVE, SENTENCE SWITCH: PASSIVE TO ACTIVE, LIST SWITCH: LEFT TO RIGHT, AND LIST SWITCH RIGHT TO LEFT. THE SIX MOTION PARAMETERS WERE INCLUDED AS REGRESSORS OF NO INTEREST. SECOND-LEVEL GROUP ANALYSES WERE THEN PERFORMED. THE VALUES FROM THE EXPERIMENTAL CONTRASTS FROM EACH SUBJECT AND CONDITION WERE ENTERED INTO A MIXED-EFFECTS ANALYSIS WITH SUBJECTS AS RANDOM VARIABLES USING AFNI’S 3DMEMA FUNCTION. WE TESTED THE FOLLOWING CONTRASTS: SENTENCE VS. LIST (**STRUCTURE**), active vs. passive (**COMPLEXITY**), and switch vs. standard (**PERTURBATION**). Because we were particularly interested in switch effects for the sentence condition, we examined the effects of PERTURBATION for sentences and lists separately in addition to the interaction of STRUCTURE and PERTURBATION. We corrected for multiple comparisons though Monte Carlo simulation using AFNI’s 3dClustSim function to hold the family-wise error (FWE) rate to less than 0.05. We estimated smoothness in the data from the residual error time series for each subject’s first-level analysis using AFNI’s 3dFWHMx function. These estimates were averaged across participants for input to 3dClustSim (simulations were restricted to in-brain voxels). Activations were considered significant with a per-voxel threshold of *p* < 0.001 (one-tailed) and a cluster size threshold of 610 mm^3^ (39 voxels).

### ROI Analyses

Given the extensive literature documenting a relationship between Broca’s area, the ATL, and sentence processing, we performed ROI analyses on these regions. We extracted percent signal change values within structural ROIs for the left and right ATL, Broca’s area, and the right hemisphere homolog of Broca’s area, the right inferior frontal gyrus (RIFG) and ran statistical analyses. For Broca’s area and the RIFG, we used templates in Talairach space for BA44 and BA45 provided by AFNI based on the cytoarchitectonic probability maps of Amunts et al. (1999). We included every voxel in each map and combined both maps together to form a single mask for Broca’s area and a single mask for the RIFG. The relevant functional regions of interest for the ATL do not align well to probability maps based on cytoarchitectonics; we constructed left and right ATL ROIs based on coordinates reported in the neuroimaging literature. We obtained the center of mass coordinates reported by Rogalsky and Hickok (2009) for the sentence > list contrast in the left and right ATL, and created spheres with radius 10 mm around the coordinates. We averaged across all voxels within each ROI and analyzed the average percent signal change values across the entire estimated timecourse. We first analyzed the effect of COMPLEXITY (passive > active) within each ROI with paired *t*-tests. We then collapsed our analyses across constructions in the sentence conditions and direction in the list conditions, resulting in 2 × 2 ANOVAs for each ROI (STRUCTURE x PERTURBATION).

## Results

### Behavioral Performance

**FIGURE 3:**
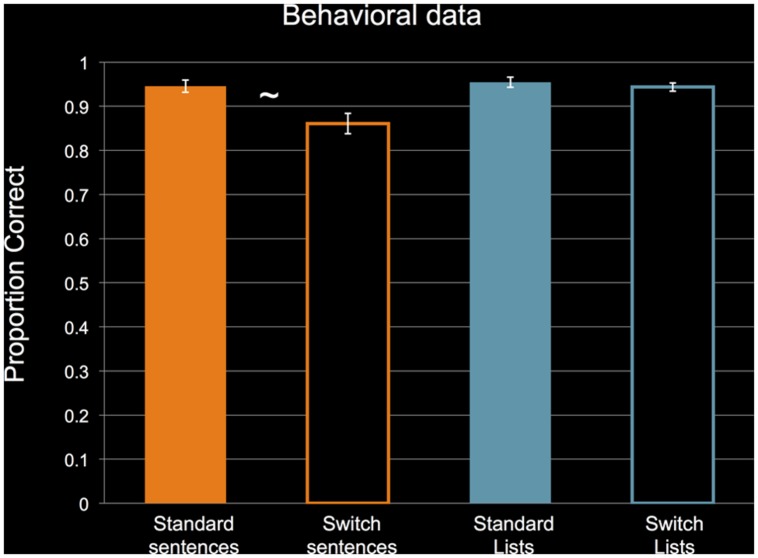
**Performance by subjects in the behavioral pre-scan training session.** *N* = 14. Data are collapsed across orientation of cue, and collapsed across constructions in the sentence conditions and right/left arrow in the list conditions. Analysis revealed a significant main effect of STRUCTURE and a significant interaction between STRUCTURE and PERTURBATION. ∼: marginally significant simple effect of PERTURBATION for sentences (*p* = 0.058) at *p* < 0.025. Error bars indicate standard error of the mean. See text for details of statistical analyses.

To reiterate, we only collected behavioral data during the behavioral training session before the fMRI session. **Figure 3** shows the behavioral performance of the 14 subjects for whom we collected data. For non-switch standard trials, subjects performed near ceiling for the sentence and list conditions. The clear outlier is the sentence switch condition. Even though subjects’ performance dropped during switch sentence trials, their performance was still above 80%, indicating that they could successfully perform the task. A 2 × 2 ANOVA revealed a significant main effect of STRUCTURE, *F*(1,13) = 5.282, *p* = 0.039, η^2^ = 0.289, no significant main effect of PERTURBATION, *F*(1,13) = 3.232, *p* = 0.095, η^2^ = 0.199, and a significant interaction, *F*(1,13) = 5.353, *p* = 0.038, η^2^ = 0.292. Follow-up two-tailed *t*-tests (α = 0.025) revealed a marginally significant effect of PERTURBATION for sentences, *t*(1,13) = 2.077, *p* = 0.058, Cohen’s *d* = 0.555, and no effect of PERTURBATION for lists, *t*(1,13) = 0.668, *p* = 0.516, Cohen’s *d* = 0.169. These results confirm that performance was only impaired during the sentence switch condition.

### Whole-Brain fMRI Analyses

**FIGURE 4:**
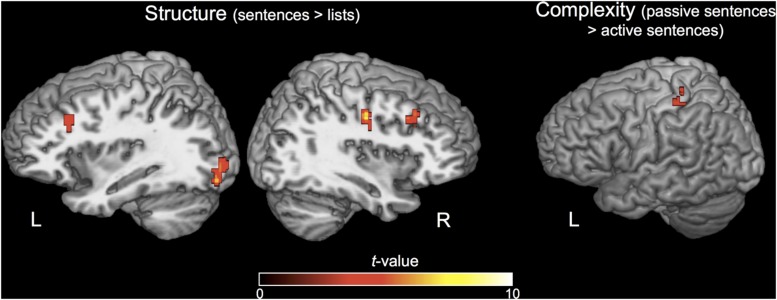
**Activations for the STRUCTURE and COMPLEXITY contrasts.** *N* = 20. Activations are displayed on a Talairach template brain and cluster-corrected for multiple comparisons at FWE *p* < 0.05, individual voxel threshold *p* < 0.001 (one-tailed), cluster size threshold 610 mm^3^.

The whole-brain contrasts of STRUCTURE and COMPLEXITY did not reveal activation in the ATL or Broca’s area. The effect of STRUCTURE (sentences > lists) revealed increased activation for sentences in left visual cortex, right precentral gyrus, right postcentral gyrus, and bilateral middle frontal gyrus (**Figure 4**). The effect of COMPLEXITY (passive > active sentences) revealed one cluster in the left postcentral gyrus (**Figure 4**). See **Table 1** for Talairach coordinates for each significant cluster of activation for these contrasts.

**Table 1:** Effects of STRUCTURE and COMPLEXITY.

Region	Hemisphere	*x*	*y*	*z*	Cluster size (mm^3^)
**Effect of structure**					
Middle frontal gyrus	Right	30	19	37	891
Inferior occipital gyrus	Left	-29	-86	-5	844
Middle frontal gyrus	Left	-27	24	33	813
Precentral gyrus	Right	38	-13	37	750
**Effect of complexity**					
Postcentral gyrus	Left	-32	-36	51	641


**N* = 20. FWE cluster-corrected *p* < 0.05; individual voxel threshold *p* < 0.001, cluster size threshold 610 mm^3^. Coordinates reflect the center of mass of each significant cluster. Coordinates are reported in Talairach space.*

**FIGURE 5:**
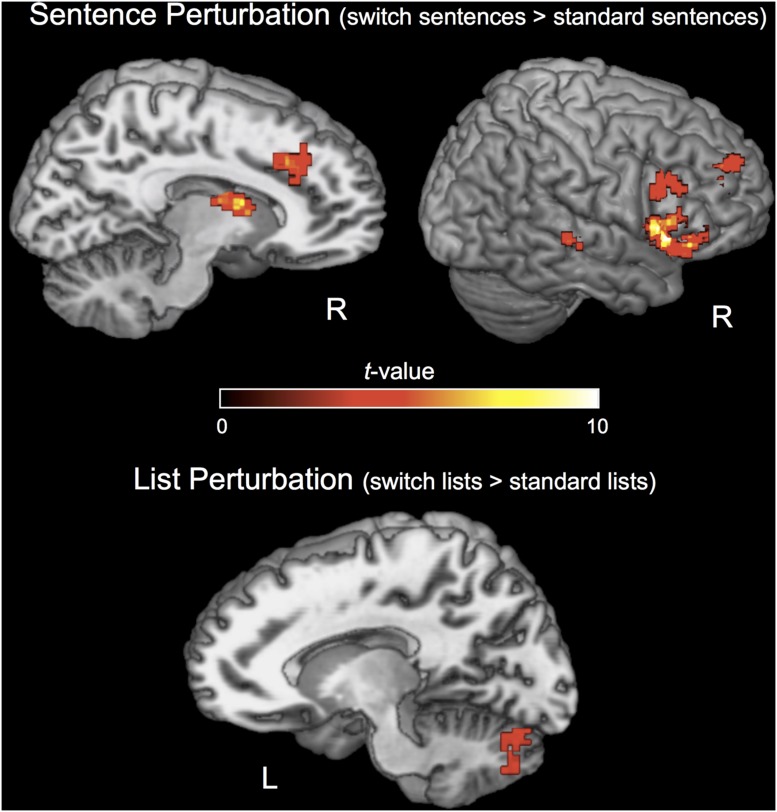
**Activations for the PERTURBATION contrasts.** *N* = 20. Activations are displayed on a Talairach template brain and cluster-corrected for multiple comparisons at FWE *p* < 0.05, individual voxel threshold *p* < 0.001 (one-tailed), cluster size threshold 610 mm^3^.

The effect of PERTURBATION in the sentence condition (sentence switch > sentence control) revealed increased activation during the switch condition in a network including areas typically found for experiments of response selection/inhibition as in the Go/No-Go task (Simmonds et al., 2008; Swann et al., 2009). The GO/No-Go task requires subjects to inhibit a planned motor response when a “stop” signal appears, as well in areas found for perturbation in low-level motor control (Diedrichsen et al., 2005; Tourville et al., 2008; **Figure 5**). Particularly strong activation was observed in the right IFG and anterior insula that has been shown to be involved in “stopping,” or the cancelation of a planned response (Aron et al., 2003, 2014). Activations for this contrast also included the supplementary motor area (SMA), pre-SMA, basal ganglia (right caudate nucleus), left inferior parietal cortex, right STS, and right IFG/MFG (**Figure 5**). The effect of PERTURBATION in the list condition (switch lists > standard lists) revealed one cluster in the left cerebellum (**Figure 5**, bottom). See **Table 2** for Talairach coordinates for each significant cluster of activation for these contrasts.

**Table 2:** Effects of PERTURBATION.

Region	Hemisphere	*x*	*y*	*z*	Cluster size (mm^3^)
**Effect of perturbation in sentences**					
Inferior frontal gyrus/anterior insula	Right	41	18	2	7,297
Superior frontal gyrus	Right	24	45	25	1,969
Medial frontal gyrus	Right	7	27	33	1,531
Superior temporal sulcus	Right	52	-28	1	844
Caudate nucleus	Right	11	1	14	813
Middle frontal gyrus	Right	52	17	28	766
**Effect of perturbation in lists**					
Cerebellum	Left	-8	-74	-26	1,078


**N* = 20. FWE cluster-corrected *p* < 0.05; individual voxel threshold *p* < 0.001, cluster size threshold 610 mm^3^. Coordinates reflect the center of mass of each significant cluster. Coordinates are reported in Talairach space.*

The interaction contrast of PERTURBATION with STRUCTURE did not reveal any significant clusters when cluster-corrected for multiple comparisons, suggesting that there was a similar switch effect across the sentence and list conditions in the brain, although the separate contrasts for these conditions activated different sets of areas.

### ROI Analyses

For the ROI analyses, based on our expectations from the literature, we separately examined the effect of COMPLEXITY (passive > active sentences) using a one-way *t*-test and performed a 2 × 2 ANOVA of PERTURBATION (switch vs. control) and STRUCTURE (sentences vs. lists).

There was no effect of COMPLEXITY (passive > active) for any of the ROIs (all reported tests are one-tailed *t*-tests). Broca’s area: *t*(1,19) = -0.059, *p* = 0.477; RIFG: *t*(1,19) = 0.069, *p* = 0.473; left ATL: *t*(1,19) = 1.746, *p* = 0.952; right ATL: *t*(1,19) = 0.799, *p* = 0.783. The high *t*-value of the left ATL indicates that there was a possibility of higher activation for *active* – less complex – sentences.

**FIGURE 6:**
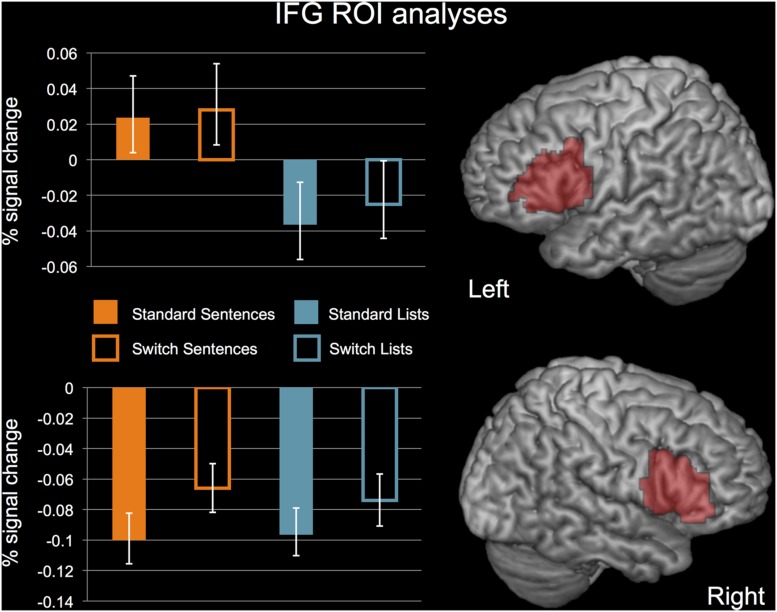
**ROI analyses for Broca’s area and the RIFG.** *N* = 20. Error bars indicate standard error of the mean. See text for details of statistical analyses.

In Broca’s area, there was no significant effect of STRUCTURE, *F*(1,19) = 1.443, *p* = 0.244, or PERTURBATION, *F*(1,19) = 0.714, *p* = 0.408, and no significant interaction, *F*(1,19) = 0.164, *p* = 0.408. In the RIFG, there was no significant effect of STRUCTURE, *F*(1,19) = 0.005, *p* = 0.946, a significant effect of PERTURBATION, *F*(1,19) = 13.541, *p* = 0.002, and no significant effect of the interaction, *F*(1,19) = 0.663, *p* = 0.426. Activations for each of these conditions in Broca’s area and the RIFG are displayed in **Figure 6.**

**FIGURE 7:**
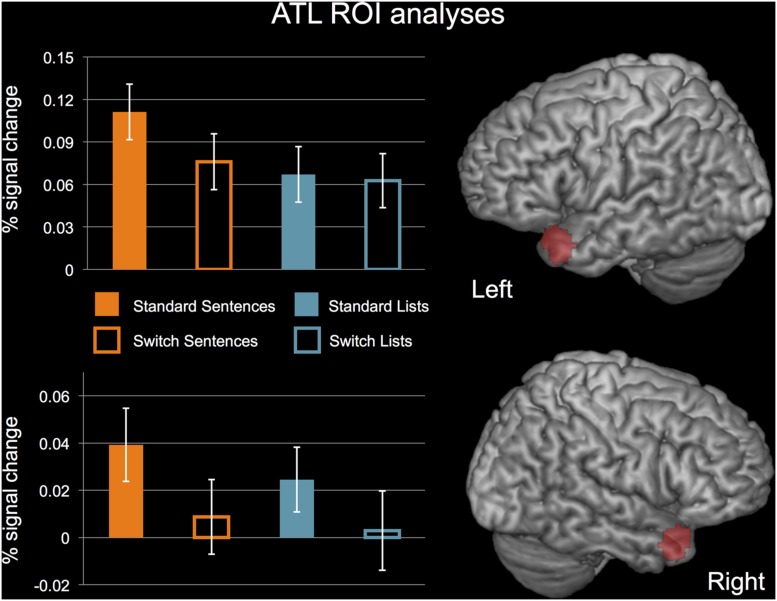
**ROI analyses for the left and right ATL.** *N* = 20. Error bars indicate standard error of the mean. See text for details of statistical analyses.

Both ATL regions showed a significant main effect of PERTURBATION (reduced activity for perturbation), no main effect of STRUCTURE, and no interaction. Left ATL: STRUCTURE, *F*(1,19) = 0.597, *p* = 0.449; PERTURBATION, *F*(1,19) = 6.963, *p* = 0.016; interaction, *F*(1,19) = 2.820, *p* = 0.110. Right ATL: STRUCTURE, *F*(1,19) = 0.123, *p* = 0.729; PERTURBATION, *F*(1,19) = 13.161, *p* = 0.002; interaction, *F*(1,19) = 0.396, *p* = 0.537. Activation for each of these conditions in left and right ATL ROIs are displayed in **Figure 7.** While the test of the interaction between STRUCTURE and PERTURBATION in the left ATL was not significant, it should be noted that this effect trended toward significance.

## Discussion

We performed a novel investigation in the effort to understand the neural bases of syntax: a constrained speech production task, including two different sentence constructions (active and passive), unstructured lists, and a “syntactic perturbation” paradigm. One goal was to probe the response profile of the traditional candidates for syntactic processing and their right hemisphere homologs with this novel PERTURBATION paradigm and a contrast of STRUCTURE (sentences > word lists). We also included a secondary contrast of COMPLEXITY (passive > active sentences) to determine if this effect previously found for comprehension in Broca’s area extended to production. Finally, another goal of the experiment was exploratory – to determine whether networks outside of the traditional candidate regions for syntax would activate to syntactic perturbation. We will first focus our discussion on the activation profiles of Broca’s area and the ATL. Following this we discuss the effects we obtained for the whole-brain contrasts, particularly the activation we obtained for PERTURBATION in the right IFG and the potential role this region plays in sentence processing.

### The Activation Profile of Broca’s Area

The domain-general hypotheses of Broca’s area suggest that this region underlies a non-syntactic mechanism during sentence processing, either resolving representational conflict through cognitive control (Novick et al., 2005) or providing working memory resources (Rogalsky and Hickok, 2011). The lack of effects for our PERTURBATION contrast in this region is consistent with these accounts, and contrary to the expectations of a region involved in syntax, as perturbation was expected to tax syntactic processing.

We also did not observe a significant effect of STRUCTURE in Broca’s area. Previous work has shown that this contrast is observable in small subregions directly adjacent to regions that do not show this contrast (Hickok and Rogalsky, 2011; Fedorenko et al., 2012a). Our structural ROIs may have contained sentence-selective and non-selective subregions, thus weakening our power to detect effects of STRUCTURE. Regardless, the contrast was clearly not robust, and combined with the fact that the PERTURBATION contrast did not approach significance in this region speaks against a syntactic function.

We did not replicate previous findings for passive > active sentences in Broca’s area in comprehension (Ye and Zhou, 2009; Mack et al., 2013). Hypotheses of Broca’s area function in sentence processing should take this disparity into account, while noting that the task constraints of our study may have substantially reduced our ability to detect activation differences between these constructions.

### The Activation Profile of the Anterior Temporal Lobe (ATL)

Our whole-brain analysis did not reveal any effects of COMPLEXITY and STRUCTURE in the left or right ATL. However, the ROI analysis did reveal a PERTURBATION effect for the ATL bilaterally – *decreased* activity for perturbation. We attribute the null effect of STRUCTURE and the decreased activity for PERTURBATION to a semantic rather than syntactic function of the ATL and decreased attention to semantic content in our study.

Our ROI plots showed less activity for switch sentences than for natural sentences, which reduced the sensitivity of our analyses to detect a main effect of STRUCTURE. This reduction can be explained by decreased attention to the semantic content of the stimulus for switch trials. Rogalsky and Hickok (2009) showed that attention substantially affects activation to semantic content in the ATL. The demanding nature of our task may have distracted subjects away from the semantic content of the sentences, reducing the difference in semantic processing between lists and sentences. Our stimuli also had limited semantic content generally. We used proper nouns instead of common nouns (e.g., *Mary* instead of *the acrobat*), and simplistic line drawings devoid of detail rather than pictures of actual people engaging in action as used in other studies (e.g., Menenti et al., 2011; Segaert et al., 2012). Future studies seeking to obtain effects of structure in the ATL during speech production should enrich the semantic nature of the materials and choose a task that does not require heavy attentional demands.

The decreased activity for PERTURBATION is contrary to the expectations of a region involved in syntax, but compatible with a role for semantics. Any effect of PERTURBATION would presumably increase demands on syntactic structure building, rather than decrease them. The increased attentional demands of switching syntactic structures, however, likely reduced attention to the semantic content of the sentences, accounting for a reduction of activity in the ATL as discussed above.

The major piece of data in support of a basic syntactic function of the ATL is the observation that the structural effect in the ATL can be found for sentences with the content words replaced by non-words, retaining the structural “feel” but with greatly impoverished semantic content (i.e., jabberwocky stimuli; Mazoyer et al., 1993; Humphries et al., 2006; Rogalsky et al., 2011). However, this effect is much less robust than for full sentences, with some studies failing to observe it at all (Pallier et al., 2011; Fedorenko et al., 2012c). Future research could determine the source of these discrepancies, including testing the notion that there may be a functional-anatomical subdivision within the ATL between syntactic and semantic processing (Rogalsky and Hickok, 2009).

### Whole-Brain Contrasts of Complexity and Structure

We first discuss the whole brain contrasts of COMPLEXITY and STRUCTURE. The whole-brain contrast of COMPLEXITY revealed one significant cluster in the left post-central gyrus. Since passive sentences are longer than active sentences, requiring additional articulation, this cluster likely reflects the increased motor speech output and corresponding somatosensory input rather than any core linguistic function. The whole-brain contrast of STRUCTURE only revealed activity in visual cortex and bilateral superior frontal areas. These regions have been previously associated with visual attention (Kastner and Ungerleider, 2000; Corbetta and Shulman, 2002). This suggests that demands on visual attention were stronger during the sentence condition than during the list condition, which is supported by the behavioral data.

The lack of additional effects in language-related regions for these contrasts deserves explanation. We have already discussed Broca’s area and the ATL; other language-related areas that are typically activated by this contrast include the left posterior temporal lobe and the angular gyrus (Bedny et al., 2011; Pallier et al., 2011; Fedorenko et al., 2012c). The difference between our results and previous studies cannot be attributed solely to the differences between production and comprehension; several production studies have revealed effects in these areas (Menenti et al., 2011, 2012; Segaert et al., 2012, 2013). As discussed in the introduction, the structurally simple and short sentences that we used minimized demands on working memory and cognitive control, and our stimuli did not encourage rich semantic processing. It may be the case that effects in these language-related regions are due to these processes. Previous research points to a role for the posterior temporal lobe in working memory and cognitive control (Hickok et al., 2003; Glaser et al., 2013) and the angular gyrus in semantic processing (Binder et al., 2009; Price et al., 2015), consistent with this speculation.

### Syntactic Perturbation Reveals a Network for Response Selection, Action Inhibition, and Motor Control

While syntactic PERTURBATION did not activate traditional language areas of the left hemisphere, it did activate other brain regions, including medial frontal areas (SMA, pre-SMA), the right caudate nucleus, the right posterior STS, the right IFG, and the right anterior insula. These are regions that have been reported in studies of perturbation and motor control in other domains (Diedrichsen et al., 2005; Suminski et al., 2007; Tourville et al., 2008) and studies of response selection/action inhibition implementing go/no-go designs (Simmonds et al., 2008). The list PERTURBATION contrast activated only the cerebellum. This disparity of results between the sentence and list conditions must be treated carefully, as the interaction contrast did not reveal a significant statistical interaction between STRUCTURE and PERTURBATION in any regions. This suggests that there were similar activation patterns for both conditions, but that the effect was somewhat stronger in the sentence condition.

The activation of the right caudate nucleus is consistent with the suggestion that the basal ganglia are involved in syntactic operations (Lieberman, 2001; Ullman, 2004). However, we do not believe that this activation in our study reflects syntax. This is because the right basal ganglia are part of a larger network that is strongly implicated in stopping, discussed below.

While RIFG activation is sometimes reported for syntactic manipulations (Embick et al., 2000; Friederici et al., 2000; Meyer et al., 2000; Fiebach et al., 2005; Tyler et al., 2010), it is not common for experiments of basic sentence processing, and the aphasia literature does not support a strong association between deficits in sentence processing and the RIFG (Damasio, 1992; but see Caplan et al., 1996). The effect of PERTURBATION in this region therefore likely reflects non-syntactic mechanisms. The operative mechanism may be action inhibition, or “stopping,” which has been attributed specifically to the RIFG in conjunction with the other areas activated by the PERTURBATION contrast (Aron et al., 2003, 2014). Under this hypothesis, the RIFG operates as a “brake.” We can apply this braking hypothesis to the current study through reverse inference. After subjects planned to produce a sentence with a given sentence construction, on switch trials they utilized the brake to inhibit this plan. When subjects planned to produce a list of words, they also relied on the brake, but less so.

Our study provides insight into a surprisingly large amount of previous studies of syntax and sentence comprehension that report activation of the RIFG. Such studies can be divided into two groups: studies of complex/non-canonical sentence constructions and garden-path sentences (Meyer et al., 2000; Fiebach et al., 2005; Grewe et al., 2006; Bornkessel-Schlesewsky et al., 2012; Chan et al., 2012), and studies involving syntactic violations (Embick et al., 2000; Moro et al., 2001; Ben-Shachar et al., 2003; Friederici et al., 2003, 2006; Bahlmann et al., 2008). The fact that our task explicitly involved stopping suggests that this mechanism may account for RIFG activations in these previous studies. When subjects process a sentence with non-canonical sentence structure or syntactic violations, they must revise their initial parse to arrive at the correct interpretation. This revision may rely on an inhibition function to quickly reject the current parse in favor of a new one. Supporting this hypothesis, Caplan et al. (1996) found that patients with right hemisphere lesions had significantly worse sentence comprehension than control subjects, particularly for complex sentence constructions (although these effects were not as strong as in patients with left hemisphere lesions). Future research could further investigate the hypothesis of a “braking” function during sentence comprehension.

## Conclusion

The present study sought to implement a novel paradigm in the study of syntax and the brain: a constrained sentence production task with a perturbation paradigm applied to syntactic structure. While our activations point to a possibility of a stopping mechanism in the RIFG that facilitates structural revision, it is difficult to make any firm conclusions based on this study alone. The lack of effects for syntactic PERTURBATION and STRUCTURE in Broca’s area suggest that this region performs a non-syntactic function during sentence processing. This supports the previous body of evidence against a role for syntax in Broca’s area (Rogalsky and Hickok, 2011). Finally, we did not extend previous effects of sentences > word lists in the ATL to production, although the lack of an effect may have been due to reduced activity in this region during perturbation. This is consistent with a role for the ATL in combinatorial semantics.

## Author Contributions

WM and GH conceptualized and designed the experiment. WM created the stimuli and collected and analyzed the data. WM and GH wrote and revised the manuscript.

## Conflict of Interest Statement

The authors declare that the research was conducted in the absence of any commercial or financial relationships that could be construed as a potential conflict of interest.
